# Endoscopic Management of Portal Hypertension

**DOI:** 10.1155/2012/747095

**Published:** 2012-07-05

**Authors:** Said A. Al-Busafi, Peter Ghali, Philip Wong, Marc Deschenes

**Affiliations:** ^1^Hepatology Unit, Division of Gastroenterology, Royal Victoria Hospital, McGill University Health Center, Montreal, QC, Canada H3A 1A1; ^2^Gastroenterology and Hepatology Unit, Department of Medicine, College of Medicine and Health Sciences, Sultan Qaboos University, P.O. Box 35, 123 Muscat, Oman

## Abstract

Cirrhosis is the leading cause of portal hypertension worldwide, with the development of bleeding gastroesophageal varices being one of the most life-threatening consequences. Endoscopy plays an indispensible role in the diagnosis, staging, and prophylactic or active management of varices. With the expected future refinements in endoscopic technology, capsule endoscopy may one day replace traditional gastroscopy as a diagnostic modality, whereas endoscopic ultrasound may more precisely guide interventional therapy for gastric varices.

## 1. Introduction

The most common cause of portal hypertension (PH) is liver cirrhosis, and this term was first introduced by Gilbert and Carnot in 1902 to describe a clinical entity characterized by ascites, splenomegaly, and variceal bleeding [1]. The development of PH in cirrhosis marks a milestone in the natural history of the disease as its complications range from the development of gastroesophageal (GE) varices with or without bleeding, ascites, hepatorenal syndrome, and hepatic encephalopathy. The hepatic venous pressure gradient (HVPG), measured as the difference between the wedged (portal vein) and the free hepatic venous pressures (inferior vena cava), becomes increased over the normal value of 5 mmHg, and is associated with variceal bleeding when elevated above 12 mmHg [2]. Varices are common in patients with cirrhosis (30% and 60% of patients with compensated and decompensated cirrhosis, resp.) [3], and if left untreated, are associated with bleeding in approximately 10% and 30% at 2 years in patients with small and large varices, respectively.

Variceal bleeding is a significant cause of morbidity and mortality worldwide [3]. Despite technical and clinical advances achieved in the last 3 decades, variceal bleeding still carries a mortality of up to 15–20% at 6 weeks with each episode (ranges from 0% in Child-Pugh class A to 32% in Child-Pugh class C) [4, 5]. Nonetheless, there have been recent improvements in survival following variceal bleeding [6], attributable to advances in resuscitation and critical care, pharmacologic therapy and endoscopic treatment.

## 2. Pathophysiology of Variceal Formation****and Rupture

Variceal bleeding is the final result of a chain of events initiated by an increase in portal pressure, followed by the development of varices and subsequent progressive dilation of these varices until they rupture and bleed. The portal system and the systemic venous circulation are connected at several locations [7], with GE collaterals being the most frequent and clinically relevant. The appearance of varices in patients with compensated cirrhosis marks the transition from clinical stage 1 (1% risk of death per year) to stage 2 chronic liver disease (3.4% risk of death per year) [3]. At this juncture, the HVPG increases to more than 10 mmHg.

Variceal rupture is governed by Laplace's Law and is the end result of increasing the variceal pressure, with increased diameter of the varices and increased wall tension with reduced wall thickness [8]. The variceal wall thickness can be evaluated visually as the presence of red wale markings, reflecting areas where it is especially thin [9, 10], and is more often found with advanced Child-Pugh class. Many studies have shown that variceal bleeding does not occur if HVPG is reduced to below 12 mmHg [11].

Variceal rupture often occurs at the level of the GE junction where the varices are very superficial and thus have thinner walls [12]. In addition, the transmural pressure of the esophageal varices (EVs) is higher than in varices at other locations due to the negative esophageal luminal pressure during inspiration, resulting in higher wall tension, and risk of rupture.

## 3. Role of Endoscopy in the Diagnosis****and Grading of Varices

Varices should be sought in all patients with clinical suspicion of cirrhosis, especially if they have stigmata of chronic liver diseases for example, spider nevi, palmar erythema, splenomegaly, and ascites. Although varices can be detected using various diagnostic and imaging techniques such as ultrasound, CT, and MRI scanning, they are less precise than endoscopy.

### 3.1. Esophagogastroduodenoscopy (EGD)

**Figure 1:**
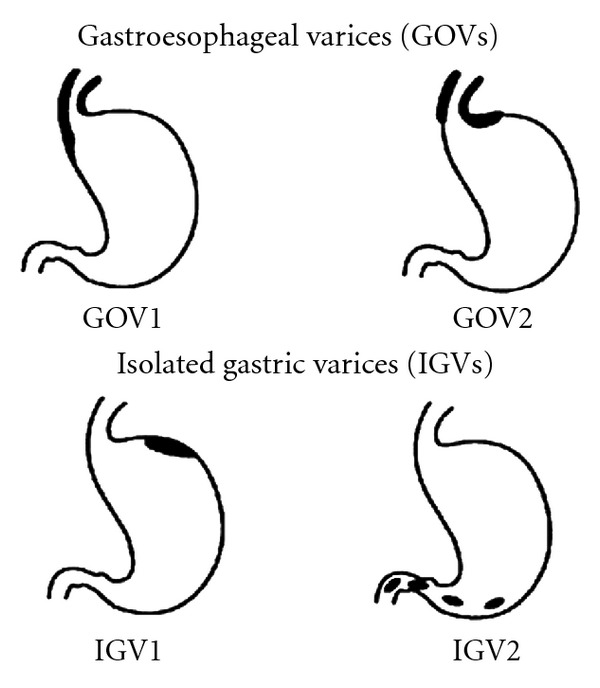
Sarin Classification of gastric varices (adapted from Sarin et al. [69]).

EGD is considered the gold standard for the diagnosis of GE varices [13]. Direct visualization is needed to assess the size and presence of high-risk stigmata of bleeding, in order to decide if prophylactic variceal banding is warranted. Examination for EV is best done during withdrawal of the scope, with the esophagus maximally insufflated with air and the stomach completely deflated in order to avoid any mucosal folds which can be interpreted as varices. GVs are generally described according to the Sarin classification and the presence or absence of red wale signs (Figure 1) [13]. EVs are usually described as in the lower, middle, or upper esophagus, and graded as small (<5 mm) or large (>5 mm) with the latter encompassing medium-sized varices when 3 grades are used (small, medium, and large) [13]. In addition, the presence of high-risk stigmata of bleeding, that is, red color signs (red wale sign and cherry red spots) must be noted.

### 3.2. Endoscopic Ultrasound (EUS)

Vascular changes within the esophagus, gastric or rectal walls can be accurately confirmed with EUS [14], but currently this modality has a limited role in clinical practice. EUS appears to perform as well as EGD for detection of clinically significant EVs [15], but is superior to EGD for detection of GV [16]. The diagnosis of GV is probably the most important clinical application of EUS in patients with PH [17], but potentially could be used to determine predictors for recurrence of varices after endoscopic obliteration, by assessing for the presence and size of paraesophageal veins [17]. EUS has no role in grading the size of esophageal varices, but in selected cases, may be of help in guiding endoscopic therapy [17–19]. Future applications may include EUS-guided direct measurement of portal pressure and transjugular intrahepatic portosystemic shunt (TIPS) placement, but to date, safety data are lacking [17].

### 3.3. Capsule Endoscopy (CE)

Current guidelines recommend screening patients with cirrhosis with EGD to detect varices [13, 20]. However, the need for sedation and invasive nature of EGD may limit acceptability by patients and adherence to screening programs [21]. Two different types of CE have been available for the evaluation of patients with portal hypertension: esophageal CE and small bowel CE. The main advantage of these diagnostic tools is that they are relatively less invasive, potentially increasing patient acceptability and adherence to screening/surveillance programs.

When esophageal CE has been compared with EGD, its performance in recognizing the presence and the size of EVs was good, but results have varied greatly across studies, and better designed trials are needed [21]. Esophageal CE has some limitations related to cost, absence of a reliable variceal size grading system, and need for specialized equipment. Currently, it can only be recommended in patients unable or unwilling to have an EGD [22]. In other studies for portal hypertensive gastropathy (PHG), esophageal CE showed sensitivity (from 74%–100%) and specificity (from 17%–83%) [22] when compared to EGD.

In the past few years, several studies have been published concerning the use of small bowel CE for detection of portal hypertensive enteropathy (PHE). The prevalence of PHE is higher than previously reported [22], but its role in causing chronic blood loss or anemia remains uncertain. CE was able to identify potential sources of bleeding in 89.5% of patients and active bleeding sites in 15.8%. Based on these findings, small bowel CE could have diagnostic utility in patients with PH and chronic anemia to identify obscure sources of bleeding [22, 23].

## 4. Role of Endoscopy in Primary Prophylaxis**** of Variceal Bleeding

**Figure 2:**
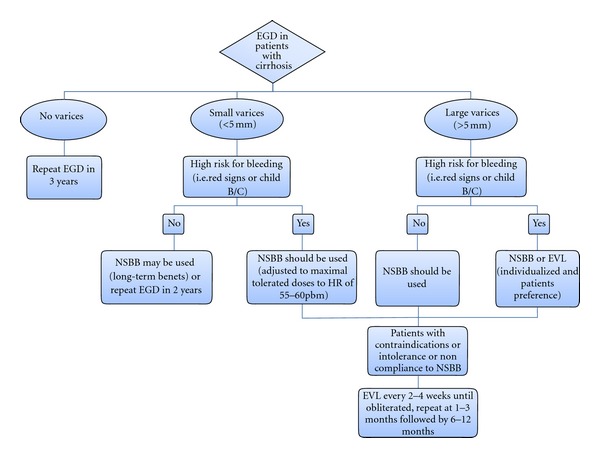
Algorithm for screening for esophageal varices and primary prophylaxis of variceal bleeding in cirrhotic patients. EGD indicates esophagogastroduodenal endoscopy; NSBB: nonselective beta blockers; EVL: endoscopic variceal ligation; HR: heart rate.

The reported risk of bleeding from GE varices in patients with cirrhosis at 1 year varies widely (ranges from 6%–76%) [10], likely reflecting the heterogeneity of the patient population. Therefore, it is important to perform EGD to identify high-risk patients who could benefit from prophylaxis for first variceal bleeding (Figure 2).

Debate exists between a pharmacologic or endoscopic approach as the best method of primary prophylaxis [13, 24]. Pharmacotherapy consists of nonselective beta blockers (NSBBs), which have systemic effects to reduce portal pressure, whereas endoscopic therapy with endoscopic variceal ligation (EVL) acts locally and has no effect on portal pressure or its evolution. Endoscopic sclerotherapy (ES) has generally been abandoned because of inconsistency of results across trials and higher morbidity and mortality than EVL [13, 24, 25].

Both NSBB and EVL are superior to no treatment for the prevention of a first variceal hemorrhage. NSBB are indicated in patients with cirrhosis and small EV with high-risk criteria for bleeding (presence of red signs or CPC B/C). In contrast, their long-term benefit in other patients with small varices has not been established [13, 24]. NSBBs or EVL as first-line therapy for primary prophylaxis of bleeding in patients with cirrhosis and large EVs with or without high-risk criteria for bleeding has been the subject of several meta-analyses [24] (Figure 2). Both modalities are effective in minimizing the risk of a first bleeding episode in patients with cirrhosis and large EV, independently of the presence of red signs. Some data suggest that EVL may be more effective in preventing first bleeding [24, 26] and is more acceptable by physicians and patients [27], but there is no benefit with regard to mortality and carries with it procedure-related complications [26]. Moreover, EVL is more expensive, requires specialized staff and cannot prevent bleeding from PHG. In contrast, NSBBs are effective, cheap, and have a more favorable safety profile. Furthermore, NSBB might have a potentially favorable effect on other PH-related complications such as spontaneous bacterial peritonitis (SBP) [24, 28].

NSBBs are the therapy of choice in patients with large EVs with no high-risk criteria for bleeding, and EVL should be considered in patients with contraindications, intolerance or noncompliance to NSBB [13].

The routine use of NSBB in patients with advanced cirrhosis has been called into question based upon a prospective study of 151 patients with cirrhosis and refractory ascites [29]. Median survival was significantly longer in patients who did not receive propranolol versus those who did (20 versus 5 months). However, more studies are needed to establish if NSBB exert different effects on different subsets of patients with cirrhosis. While waiting for the results of such studies, patients with ascites who are on NSBB should be monitored closely, and consideration should be given to discontinuing NSBB when either sepsis or HRS develop [30].

In addition to ES, other approaches to primary prophylaxis that are not recommended include nitrates (either alone or in combination with NSBB), shunt therapy, and combination therapy with NSBB and EVL [13, 20].

Based on the current evidence, EGD surveillance is recommended in patients with no varices (every 3 years) or with small varices not receiving prophylaxis (every 1-2 years), in order to detect newly formed large varices [13]. Patients with decompensated cirrhosis should have EGD at the time of diagnosis and annually thereafter. Routine follow-up EGD is not necessary for patients who receive NSBB but may be performed when clinical picture dictates.

## 5. Endoscopic Management of Acute Variceal Bleeding (AVB)

Acute variceal bleeding in patients with cirrhosis indicates decompensation and a high-risk of death [3]. Management of AVB should aim both at controlling bleeding and at preventing early rebleeding, which is particularly common within the first week and is associated with increased mortality [31]. The management of the AVB is a multistep process that includes the initial assessment of the patient, effective resuscitation, timely diagnosis, control of bleeding, and prevention of early rebleeding and complications such as infection, hepatorenal syndrome, or hepatic encephalopathy. Complicated cases may require a multidisciplinary approach involving a gastroenterologist, intensivist, general surgeon and interventional radiologist. It has been previously shown that about two-thirds of deaths in which bleeding is the precipitating cause occur within 24 hours of the onset of bleeding, thus emphasizing the need to act quickly and decisively as soon as the patient reaches the hospital [32].

**Figure 3:**
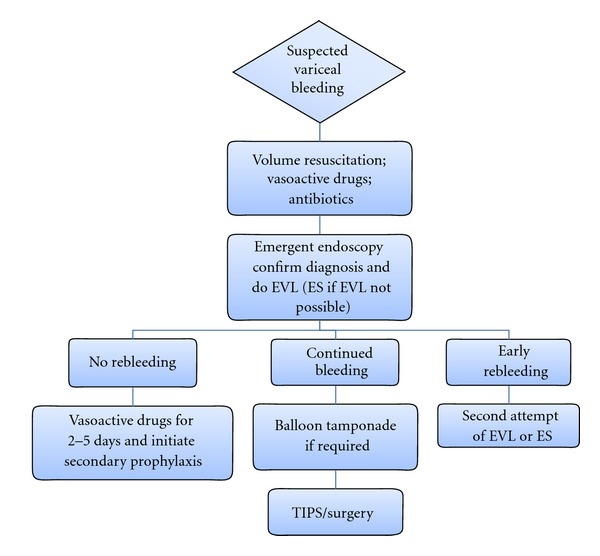
Algorithm for management of acute variceal bleeding. EVL indicates endoscopic variceal ligation; ES: endoscopic sclerotherapy; TIPS: transjugular intrahepatic portosystemic shunt.

The initial management includes appropriate volume resuscitation, blood transfusion to keep hemoglobin levels approximately 80 g/L, antibiotic prophylaxis, and endotracheal intubation in selected cases (Figure 3) [13]. Vasoactive drugs (terlipressin; somatostatin or its analogues octreotide and vapreotide) should be initiated as soon as variceal bleeding is suspected and continued for up to 5 days after diagnosis is confirmed [13].

Emergency EGD, performed within the first 12 hours of admission, is one of the cornerstones of management as it confirms diagnosis and is therapeutic. It is known that about 25–30% of bleeds in cirrhotic patients are of nonvariceal origin, mainly peptic ulcer and PHG [8]. In addition, when endoscopy is done early, active bleeding is found in 39–44% of patients, with 33–44% showing signs of recent bleeding (clots or “white nipple” on varices) [33], but no sign of active or recent hemorrhage in the remaining 12–28% [8]. There are 2 endoscopic methods available for AVB: endoscopic sclerotherapy (ES) and endoscopic variceal ligation (EVL).

**Endoscopic Sclerotherapy (ES)**

ES was first described in 1938 by Crafford and Frenckner using operative rigid endoscopes with patients under general anesthesia [34]. Currently, ES is relatively easy to perform by fiberoptic endoscopy using flexible catheters with a short needle tip (23 or 25 gauge). Sclerosants are injected into the variceal lumen (intravariceal) or adjacent to it (paravariceal) with rapid thrombus formation. Both intravariceal and paravariceal injections have been associated with equally good outcomes [35]. The outcomes are also similar regardless of the type of sclerosant used [36], the volume injected, or frequency of sessions [37].

Compared to EVL, the advantages of ES are its ease of use, quick assembly, and lack of a need to withdraw and reinsert the endoscope. However, ES is associated with more complications than EVL, such as chest pain, fever, dysphagia, pleural effusion, and perforation [38, 39]. Rarer complications include esophageal strictures, mediastinitis, chylous effusion, pneumonia and bacteremia leading to SBP and distal abscesses [38, 40]. Esophageal ulcers are common and may cause bleeding in 20% of patients [38]. A recent Cochrane meta-analysis showed that ES was not superior to the vasoactive drugs in terms of control of bleeding, rebleeding, and mortality [41].

**Endoscopic Variceal Ligation (EVL)**

The first reports of EVL appeared in 1988 by Stiegmann et al. [42], and the procedure was developed as an alternative to ES for treatment of AVB. The introduction of multiband devices, which allow the placement of 4–10 bands at a time, has made the technique easier to perform, avoiding the use of overtubes and their related complications. Endoscopic variceal ligation causes occlusion of the varix and then thrombosis with ischemic necrosis of the mucosa. When the bands fall off a few days later, a superficial ulceration is left which eventually scars [43], making subsequent redevelopment of varices more difficult. Compared to ES, a meta-analysis of 7 randomized controlled trials (RCTs) showed a tendency toward benefit of EVL in the initial control of bleeding, recurrent bleeding, side effects, need for fewer endoscopic treatments, and survival [39]. Interestingly, HVPG transiently decreases after EVL, while it increases after ES [44]. Therefore, EVL has become the treatment of choice for AVB, although ES can be used in patients in whom EVL is technically difficult, for example, in treating patients with AVB where there is marked difficulty in visualizing the mucosa [13, 20].

Complications of EVL include chest pain and transient dysphagia which are common and respond well to oral analgesia and oral antacids. Superficial esophageal ulcers are frequent, but seldom bleed. Other potential complications such as massive bleeding from variceal rupture, esophageal perforation, and esophageal strictures [45] are fortunately rare. Additionally, EVL may cause worsening of and/or appearance of PHG [46].

**Combination Therapy**

Combination of vasoactive drugs plus EVL has been proposed as the standard of care for AVB [13, 20]. A meta-analysis of 8 trials involving 939 patients demonstrated that compared to endoscopic therapy alone (ES or EVL), endoscopic and vasoactive drugs (octreotide, somatostatin, or vapreotide) therapy improved the initial control of bleeding and 5-day hemostasis without differences in severe side effects or mortality [47].

Other studies have looked at combining EVL and ES in order to speed variceal eradication, reduce the likelihood of rebleeding [48], and reduce the incidence of recurrent varices [49]. A meta-analysis of 7 RCTs by Singh et al. noted that combination therapy offered no advantage over EVL alone in the control of bleeding varices, prevention of rebleeding or reducing mortality [50]. In addition, a significantly higher incidence of esophageal stricture was seen with combination therapy. Several variations in the types of sclerosants and the protocol for administering ES in combination with EVL have been described [51, 52].

Data on other combination therapies including EVL with thermal therapies either argon plasma coagulation (APC) [53–55] or microwave cautery are emerging [56]. However, none of these techniques has been sufficiently studied to be recommended in routine clinical practice.

**Failures of Endoscopic Therapy**

Treatment failure is defined as a failure to control AVB within 24hours, or failure to prevent clinically significant rebleeding or death within 5 days of treatment [20]. The current first-line therapy, that is, pharmacologic and endoscopic, fails to control bleeding in approximately 10–15% of patients [8, 13]. These patients are at high-risk for exsanguinating and other complications related to active bleeding. Child-Pugh class, shock at admission, presence of portal vein thrombosis, active bleeding at endoscopy, and elevated HVPG >20 mmHg have been shown to be predictive of treatment failure [8, 57].

Although a post hoc analysis of a RCT suggested that a higher dose of somatostatin (500 *μ*g/h) had significantly higher control of bleeding and better survival [48], this finding awaits confirmation by trials. A second attempt at endoscopic therapy using EVL or ES can be performed in more stable patients, for example, EVL in patients who failed ES [58]. If this is unsuccessful, more definitive therapy must be instituted with shunt therapy (surgical or TIPS))[13]. Indeed a recent RCT showed that early use of TIPS (i.e., within 72 hours after admission) in patients with AVB and at high-risk for treatment failure (i.e., Child-Pugh class C cirrhosis (a score of 10 to 13) or class B disease (a score of 7 to 9) with active variceal bleeding) was associated with significant reductions in treatment failure and in mortality [59].

Balloon tamponade can also be used in patients who failed in initial endoscopic therapy to obtain temporary hemostasis (maximum 24 hours) while preparing for more definitive therapy. Preliminary studies have described the placement of self-expanding metallic stents as an alternative to balloon tamponade for the control of refractory variceal hemorrhage [60, 61]. In these studies, the stents had a high success rate with minor complications. However, these findings must be confirmed in well-designed trials before use in clinical practice.

## 6. Role of Endoscopy in Secondary Prophylaxis of EV Bleeding

Once AVB is successfully controlled, rebleeding may occur in approximately 60% of patients if preventive measures are not taken [13]. It is, therefore, essential that patients, who survive an episode of AVB, should receive secondary prophylaxis to improve survival. The approaches recommended include NSBB, EVL, TIPS, shunt surgery, and liver transplantation [13, 20]. Combined approaches with NSBB plus EVL are considered the best option for secondary prophylaxis of variceal hemorrhage [62, 63]. In patients who are not candidates for EVL, the strategy would be to maximize portal-pressure reduction by combining NSBB plus nitrates [5]. Shunt operations or TIPS are reserved for endoscopic and medical failures [13, 20].

ES has been largely replaced by EVL and should no longer be used in the secondary prophylaxis of variceal bleeding [13]. A meta-analysis of 7 trials showed that, compared with ES, EVL reduced the rebleeding rate (odds ratio 0.46), the mortality rate (odds ratio 0.67), the rate of death due to rebleeding (odds ratio 0.49), and the development of esophageal strictures (odds ratio 0.1) [39]. Variceal obliteration was achieved in similar proportions with both techniques, but the number of treatments necessary to achieve obliteration was lower with EVL.

Combination of EVL with other endoscopic modalities to manage EVs has been a focus of research for gastroenterologists. Studies evaluating different approaches have produced heterogeneous results. Considering the available data, it appears that the addition of ES [64–66], microwaves [67], or APC [55] following variceal obliteration achieved by EVL could effectively reduce variceal recurrence. However, controlled trials are needed before they can be routinely recommended. In contrast to these findings, most studies using synchronous combination of EVL and ES during initial variceal obliteration have demonstrated decreased efficacy and a higher complication rate compared with EVL alone [68].

## 7. Endoscopic Management of Gastric Varices (GVs)

Bleeding from GV is fortunately less frequent, but generally more severe than bleeding from EV and may be technically difficult to treat [69]. In GV, the blood flow is relatively increased, and so the bleeding is often rapid and torrential. Although prospective RCTs in successful endoscopic hemostasis and obliteration of GV using different agents and techniques with improved outcome of GV bleeds have been reported, no consensus has been reached on the optimal therapy [70]. The problem is that heterogeneous types of GV including GOV1 in more than 50% subjects have been included in these trials without definite explanation or classification of the varices, making it difficult to compare with studies [70–72].

**Figure 4:**
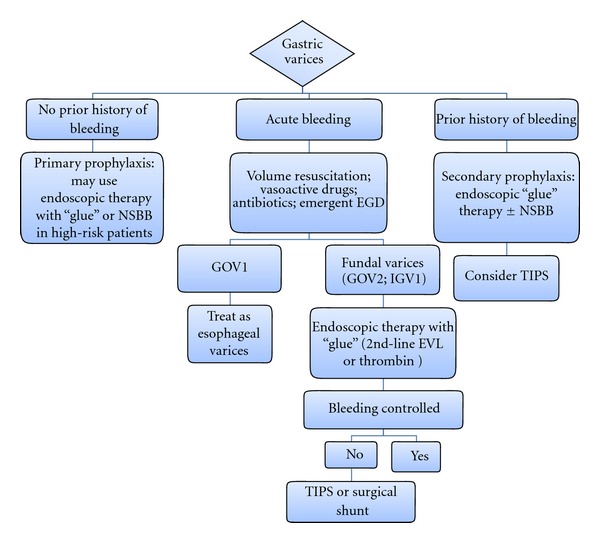
Algorithm for endoscopic management of gastric varices. NSBB indicates nonselective beta blockers; EGD: esophagogastroduodenal endoscopy; GOV: gastroesophageal varices; IGV: isolated gastric varices; EVL: endoscopic variceal ligation; TIPS: transjugular intrahepatic portosystemic shunt.

The endoscopic treatment modalities largely depend on the type of the GV (Figure 4). The Sarin classification, which categorizes GV based on their location in the stomach and their relationship with EV, is most widely used (Figure 1) [20, 69].

**Control of Acute GV Bleeding**

The literature on the endoscopic management of GV bleeding is not as clear as that for EV. Gastroesophageal varices type 1 (GOV1) constitute an extension of esophageal varices along the lesser curvature of the stomach. Therefore, they should be managed in the same way as EV. In addition, the GOV1 bleeding, hemostasis and rebleeding rate are similar to those of EV [73]. Currently, there are limited data regarding the management of bleeding from fundal varices (gastroesophageal varices type 2 (GOV2) or isolated gastric varices type 1 (IGV1)). An exception is IGV1 which are secondary to isolated splenic vein thrombosis, in which therapy consists of splenectomy. There are various endoscopic techniques of treatment for fundal varices including, ES, EVL, gastric variceal obliteration (GVO) with glue, and thrombin injection.

Compared to its efficacy for treatment of GOV1 bleeding, ES was shown by a number of studies to be ineffective for patients with fundal varices because of low rate of primary hemostasis, high rate of rebleeding and high incidence of local complications, for example, perforation and ulcer formation [70]. The reason is that there is a high volume of blood flow through GV compared to EV, resulting in the rapid escape of sclerosant into the systemic circulation.

Compared to ES or EVL, GVO with a tissue adhesive (polymers of cyanoacrylate) is more effective for acute fundal GV bleeding with a better rate of controlling the initial hemorrhage as well as lower rebleeding rate [70–72, 74–76]. Therefore, cyanoacrylate is recommended as the preferred treatment for control of bleeding from fundal GV, where it is available and with appropriate expertise [13, 20]. In the United States, it is used only in a few centers under research protocols, and its use is not approved by the United States Food and Drug Administration.

When introduced into the varix and upon contact with blood, cyanoacrylate immediately polymerizes into a firm clot leading to obliteration of the varix. Complications from cyanoacrylate injection are rare, and these include rebleeding due to extrusion of the glue cast (4.4%), sepsis (1.3%), distant emboli (pulmonary, cerebral, and splenic; 0.7%), gastric ulcer formation (0.1%), major GV bleeding (0.1%), and mesenteric hematoma associated with hemoperitoneum and bacterial peritonitis (0.1%). The complication-related mortality rate is approximately 0.5% [77]. In addition, cyanoacrylate can also be used as secondary prophylaxis for GV bleeding. In one trial, cyanoacrylate was more effective than NSBB for the prevention of rebleeding and improved survival during a median followup of 26 months [78].

The evidence for efficacy of EVL for treatment of bleeding GVs is mixed because most of the studies used small sample sizes and had predominantly patients with GOV1 or 2 [70]. However, a relatively large RCT with 2 years of followup and a greater proportion of IGV1 patients, comparing GVO with cyanoacrylate glue versus EVL in cirrhotics with acute GV bleeding, showed that both treatment arms were similar in controlling active bleeding but rebleeding was higher in EVL group [71]. Therefore, EVL is recommended to be used as an alternative option, where tissue adhesives are not available [13]. Another study has shown the successful use of elastic bands and detachable snares in controlling acute rebleeding and achieving gastric variceal eradication [79], but the cumulative variceal recurrence rate was 100% at 2 years.

Another promising alternative endoscopic therapeutic agent is the intravariceal injection of thrombin [70, 80–82]. Thrombin has not been subjected to controlled trials, but the available data have suggested its usefulness in achieving excellent initial hemostasis and in being easy and very safe to use for control of GV bleeding [70]. Further controlled trials are required before it can be universally recommended. TIPS should be considered if endoscopic therapy is not possible or after a single failure of endoscopic treatment [13].

**Primary Prophylaxis for GV Bleeding**

There are limited data on primary prophylaxis of GV bleeding [20]. In a recently published well-designed RCT with large sample size and median followup of 26 months, cyanoacrylate was found to be more effective than NSBB therapy in preventing first GV bleeding and also to improve survival in patients with high-risk GVs (GOV2 and IGV1) [83]. High-risk factors for first bleeding from GVs were of size GV >20 mm, MELD score ≥17, and the presence of PHG.

## 8. Endoscopic Management of PHG and GAVE

The mucosal changes in the stomach of patients with PH which may present with bleeding include PHG and gastric antral vascular ectasia (GAVE). These are 2 clearly distinct clinical entities with different pathophysiology, endoscopic appearance, and treatment. Portal hypertensive gastropathy, as its name indicates, is associated with PH, whereas GAVE is also found in patients without PH or liver disease. Liver failure appears to play a role in the development of GAVE but has been shown to resolve after liver transplantation [84]. PHG is typically located in the proximal stomach, whereas GAVE is typically located, as its name indicates, in the gastric antrum. PHG is primarily an endoscopic diagnosis based on the presence of red spots on a background of snakeskin mosaic pattern, whereas GAVE is endoscopically characterized by the presence of red spots without a background mosaic pattern [20].

The management of PHG is based on measures that reduce portal pressure, namely, the use of octreotide in the acute setting [85] and NSBB with iron therapy in chronic blood loss [86]. TIPS should be considered as salvage therapy in patients with recurrent bleeding despite pharmacological therapy [87]. Only one single center study of 29 patients (11 patients with PHG) has evaluated the use of endoscopic therapy of PHG with APC [88]. The APC was successful in managing bleeding and reducing transfusion requirement in this group of patients. The data are limited, and this endoscopic approach needs further evaluation by RCT, but it could be considered in patients who are transfusion-dependent in spite of NSBB and those who are not candidate for TIPS.

Specific measures to treat patients with bleeding GAVE are substantially different from those used in PHG. It does not respond to portal pressure reducing therapies, such as TIPS or shunt surgery. The mainstay of therapy in GAVE is the endoscopic ablation of the lesions. There are different endoscopic therapeutic methods which have been used in the setting of GAVE including APC, heater probe, gold probe, cryotherapy, band ligation, and laser therapy [89]. Most studies evaluating the use of APC have reported good results [88–90]. APC, which produces thermal coagulation by applying contact with mucosa, is easy to use and the risk of perforation is much lower than with laser therapy. Complications associated with this method are gastric outlet obstruction [91] and the formation of hyperplastic polyps [92]. The sessions should be repeated every 2 to 6 weeks as needed.

Other studies have evaluated the use of different drugs for example, estrogen-progesterone, thalidomide, and surgery with antrectomy, but these should be reserved for when endoscopic therapy has failed. Antrectomy has high morbidity and mortality particularly in patients with decompensated cirrhosis in whom GAVE usually presents.

## 9. Endoscopic Management of Ectopic Varices (EcVs)

Varices occasionally develop at sites other than the stomach and esophagus and come to clinical attention when they bleed. Examples are duodenal, rectal, and peristomal varices. Duodenal varices are the most prevalent and most common cause of bleeding from ectopic varices (EcVs).

Because EcVs are infrequent and account for less than 5% of all PH-related bleeding, there have been no RCTs on the management of this condition, and it is unlikely that there ever will be such a trial. The management is mainly extrapolated from the GE varices literature and a few small studies done in patients with bleeding EcV. Successful outcomes depend on local expertise, location of varices, and the technical feasibility [93]. Initial management involves hemodynamic stabilization, use of vasoactive drugs and antibiotic prophylaxis [13]. Octreotide has been shown to be effective in the control of bleeding colonic varices [94].

Endoscopy is used for both diagnosis and therapy. Most EcVs are within reach of standard endoscopy [95], and for the rest, enteroscopy might be used [96]. ES has been used successfully in controlling bleeding varices in the duodenum [97, 98], rectum [99, 100], and in stomal varices [101, 102]. However, there have been reports of cases of rebleeding of duodenal varices after ES [103], and this is probably a result of the large varices in this area, such that sclerosants fail to concentrate, thereby diminishing the obliterative effects. Cyanoacrylate glue injection has been successfully used to obliterate bleeding duodenal [104, 105], jejunal [106], and rectal varices [107].

EVL for bleeding duodenal varices is challenging because of limited visibility from the banding hood. It may be useful for temporary hemostasis but rebleeding is a problem [108, 109]. However, several cases of successful treatment of rectal varices using EVL have been reported [110, 111].

EUS can be used to better localize and differentiate ECV from other bleeding mucosal lesions [112, 113]. In patients with rectal varices, EUS is a more sensitive diagnostic study than regular endoscopy in detecting early as well as florid changes [114, 115]. Furthermore, EUS can be used to apply a sclerosant or coil embolization when adequate visualization is not possible with conventional endoscopy [116, 117]. EUS is also useful to follow up therapy of the varix after therapy.

## 10. Summary

The development of GE varices is a serious consequence of portal hypertension. Endoscopy plays an indispensible role in the management of varices including diagnosis, staging, preventing first bleeding, control of active bleeding, and preventing rebleeding. This approach has had a positive impact on patient survival. Capsule endoscopy in the future could potentially become an alternative to regular endoscopy for evaluation of the consequences of portal hypertension in the esophagus, stomach, and small bowel. Endoscopic ultrasound can be used to diagnose gastric and ectopic varices as well as to help in guiding endoscopic therapy.
